# Performance evaluation of the SMG HHV-6 Q Real-Time PCR Kit for quantitative detection and differentiation of human herpesvirus 6A and 6B

**DOI:** 10.1128/spectrum.04249-23

**Published:** 2024-03-07

**Authors:** Tae Yeul Kim, Min-Seung Park, Sun Ae Yun, Minhee Kang, Doo Ri Kim, Areum Shin, Hyun-Young Kim, Mi-Ae Jang, Ja-Hyun Jang, Min-Jung Kwon, Hee Jae Huh, Yae-Jean Kim, Nam Yong Lee

**Affiliations:** 1 Department of Laboratory Medicine and Genetics, Samsung Medical Center, Sungkyunkwan University School of Medicine, Seoul, South Korea; 2 Department of Laboratory Medicine, Kangbuk Samsung Hospital, Sungkyunkwan University School of Medicine, Seoul, South Korea; 3 Center for Clinical Medicine, Samsung Biomedical Research Institute, Samsung Medical Center, Seoul, South Korea; 4 Biomedical Engineering Research Center, Smart Healthcare Research Institute, Samsung Medical Center, Seoul, South Korea; 5 Department of Medical Device Management and Research, Samsung Advanced Institute for Health Sciences and Technology, Sungkyunkwan University, Seoul, South Korea; 6 Department of Pediatrics, Samsung Medical Center, Sungkyunkwan University School of Medicine, Seoul, South Korea; 7 Samsung Advanced Institute for Health Sciences and Technology, Sungkyunkwan University, Seoul, South Korea; Johns Hopkins Medicine, Baltimore, Maryland, USA

**Keywords:** HHV-6, PCR, SMG assay, RealStar assay

## Abstract

The aim of this study was to compare the performance of the newly developed SMG HHV-6 Q Real-Time PCR Kit (SMG assay) with the RealStar HHV-6 PCR Kit (RealStar assay). The analytical sensitivity and specificity, linearity, and precision of the SMG assay were evaluated. The clinical performance of the SMG assay was assessed and compared with that of the RealStar assay using 207 clinical specimens (HHV-6A positive, *n* = 51; HHV-6B positive, *n* = 64; HHV-6A/B negative, *n* = 92). The limit of detection of the SMG assay was 2.92 log_10_ copies/mL for HHV-6A DNA and 2.88 log_10_ copies/mL for HHV-6B DNA. The linear range was determined to be 3.40–9.00 log_10_ copies/mL for both viruses. Intra- and inter-assay variability were below 5% at concentrations ranging from 4 to 9 log_10_ copies/mL. No cross-reactivity was observed with the 25 microorganisms included in the specificity panel. The clinical sensitivity and specificity of the SMG and RealStar assays compared to in-house polymerase chain reaction and sequencing were as follows: SMG assay, 98.0% and 100% for HHV-6A DNA, respectively, and 96.9% and 100% for HHV-6B DNA, respectively; RealStar assay, 98.0% and 100% for HHV-6A DNA, respectively, and 90.6% and 100% for HHV-6B DNA, respectively. The correlation coefficients between viral loads measured by the two assays were 0.948 and 0.975, with mean differences of 0.62 and 0.32 log_10_ copies/mL for HHV-6A and HHV-6B DNA, respectively. These results demonstrate that the SMG assay is a sensitive and reliable tool for the quantitative detection and differentiation of HHV-6A and HHV-6B DNA.

**IMPORTANCE:**

Quantitative real-time PCR (qPCR) that can distinguish between HHV-6A and HHV-6B DNA is recommended for diagnosis of active infection. The SMG HHV-6 Q Real-Time PCR Kit (SMG assay) is a newly developed qPCR assay that can differentiate between HHV-6A and HHV-6B DNA; however, little is known about its performance. In this study, we assessed the performance of the SMG assay and compared it with that of a commercially available qPCR assay, the RealStar HHV-6 PCR Kit (RealStar assay). The SMG assay demonstrated excellent analytical sensitivity and specificity, precision, and linearity. Furthermore, the viral loads measured by the SMG assay were highly correlated with those measured by the RealStar assay. Our results suggest that the SMG assay is a useful diagnostic tool for quantitative detection and differentiation of HHV-6A and HHV-6B DNA.

## INTRODUCTION

Human herpesvirus 6 (HHV-6) is a double-stranded DNA virus belonging to the *Roseolovirus* genus within the Betaherpesvirinae subfamily (1). Primary infection with HHV-6 usually occurs in infancy or early childhood, and over 95% of children become infected with HHV-6 by the age of two (2, 3). After primary infection, HHV-6 establishes latency in various cell types including lymphocytes, monocytes, and neuronal cells by integrating its genome into the telomeres of host chromosomes (4–6). When chromosomal integration of the HHV-6 genome occurs in germline cells, the integrated viral genome is transmitted to subsequent generations in a Mendelian fashion. This condition, known as inherited chromosomally integrated HHV-6 (iciHHV-6), is found in approximately 1% of the general population, and individuals with iciHHV-6 characteristically have very high levels of HHV-6 DNA in blood and other specimen types, including saliva and cerebrospinal fluid (7–11). Reactivation of HHV-6 from latency commonly occurs in immunocompromised patients, particularly in hematopoietic stem cell transplant (HSCT) and solid organ transplant (SOT) recipients, resulting in severe complications such as encephalitis (12–19).

HHV-6 is divided into two subgroups, HHV-6A and HHV-6B, with an overall nucleotide sequence identity of 90% (20). Initially considered as variants of the same species, HHV-6, HHV-6A, and HHV-6B, respectively, have recently been recognized as distinct species by the International Committee on Taxonomy of Viruses, based on differences in genetic, immunological, biological, and epidemiological characteristics (21, 22). It is well known that HHV-6B is more commonly associated with human diseases than HHV-6A. Primary infection with HHV-6B is the main cause of exanthem subitum, a common childhood disease characterized by high fever and skin rash (1). Reactivation of HHV-6B frequently occurs in HSCT and SOT recipients and is associated with skin rash, cytomegalovirus (CMV) reactivation, hemorrhagic cystitis, acute graft-versus-host disease, and encephalitis (13, 14, 17, 18). In contrast, HHV-6A has not yet been definitively associated with any human disease, and its natural history is largely unknown.

The diagnosis of active HHV-6 infection is usually made by detecting HHV-6 DNA in whole blood, plasma, or serum by polymerase chain reaction (PCR) (23, 24). However, latent HHV-6 DNA and iciHHV-6 DNA can also be detected by PCR, leading to misdiagnosis of active infection and unnecessary treatment (25–27). Quantitative real-time PCR (qPCR) may be useful for distinguishing active HHV-6 infection from these cases (23, 24). Various qPCR assays for measuring HHV-6 DNA load are available, and some of them can differentiate between HHV-6A and HHV-6B DNA (28–35).

The SMG HHV-6 Q Real-Time PCR Kit (SMG assay; Hwainmedics, Seoul, South Korea) is a newly developed qPCR assay that can differentiate between HHV-6A and HHV-6B DNA. While this assay is now commercially available, it has not yet obtained Food and Drug Administration or Conformité Européenne in vitro Diagnostics (CE-IVD) approval as of the time of writing, and little is known about its performance. In this study, we assessed the performance of the SMG assay and compared it with that of the RealStar HHV-6 PCR Kit (RealStar assay; Altona Diagnostics, Hamburg, Germany), which is a commercially available qPCR assay routinely used in diagnostic laboratories.

## MATERIALS AND METHODS

### Clinical specimens

Whole blood specimens were submitted to the Molecular Microbiology Laboratory at Samsung Medical Center, Seoul, South Korea, for routine HHV-6 testing. DNA was extracted using MagNA Pure 96 (Roche Diagnostics, Rotkreuz, Switzerland), with an input volume of 200 µL and an eluate volume of 100 µL, or KingFisher Flex (Thermo Fisher Scientific, Waltham, MA, USA), with an input volume of 200 µL and an eluate volume of 50 µL, and was subjected to in-house PCR targeting the HHV-6 DNA polymerase gene. The limit of detection (LOD) of the in-house PCR was 3.05 log_10_ copies/mL [95% confidence interval (CI): 2.91–3.31 log_10_ copies/mL] for HHV-6A DNA and 3.10 log_10_ copies/mL (95% CI: 2.96–3.33 log_10_ copies/mL) for HHV-6B DNA. The following primer pair described by Reddy and Manna (28) was used: 5′-CTGACAGACATAAAGATGCTATCCGT-3′ (forward) and 5′-CGGGTTATTGCCGTGTGT-3′ (reverse). For PCR amplification, 1 µL of DNA template and 1 µM of each primer were added to AccuPower HotStart PCR Premix (Bioneer, Daejeon, South Korea). The cycling conditions used were as follows: initial denaturation at 95°C for 5 min, then 40 cycles of 95°C for 20 s, 60°C for 30 s, and 72°C for 30 s, followed by a final extension step at 72°C for 5 min. The PCR products were separated by agarose gel electrophoresis and visualized under ultraviolet light after staining with ethidium bromide. The PCR products showing the expected band of 207 bp on agarose gel were considered positive for HHV-6 and were further subjected to Sanger sequencing using the same primer pair as for PCR amplification and BigDye Terminator (v.3.1) Cycle Sequencing Kit (Thermo Fisher Scientific). The resulting sequences were used to differentiate between HHV-6A and HHV-6B. Residual DNA specimens remaining after routine HHV-6 testing were deidentified and stored at –70°C until use in this study. Two hundred and seven DNA specimens (51 HHV-6A-positive, 64 HHV-6B-positive, and 92 HHV-6A/B-negative specimens) obtained from whole blood of 121 patients [male: 81, female: 40; median age: 48 (range: 0.2–80) years] between September 2017 and May 2023 were thawed and tested in parallel using the SMG and RealStar assays.

### Quantitative real-time PCR

Both the SMG and RealStar assays were performed according to the manufacturers’ instructions. Briefly, for both assays, 10 µL of sample DNA was added to 21 µL of PCR master mix, giving a total reaction volume of 31 µL. PCR was performed on the CFX96 system (Bio-Rad, Hercules, CA, USA) using the following cycling conditions: initial denaturation at 95°C for 5 min, followed by 45 cycles of 95°C for 15 s and 60°C for 30 s (SMG assay); initial denaturation at 95°C for 10 min, followed by 45 cycles of 95°C for 15 s and 58°C for 60 s (RealStar assay). In each run, four quantification standards provided in the kit (SMG assay: 2, 3, 4, and 5 log_10_ copies/µL for both viruses; RealStar assay: 1, 2, 3, and 4 log_10_ IU/µL for both viruses) were assayed to construct standard curves. HHV-6A and HHV-6B DNA loads were calculated from the standard curves and expressed as copies per milliliter (SMG assay) or international units per milliliter (RealStar assay) of whole blood. Values given as international units per milliliter were converted to copies per milliliter using the manufacturer’s conversion factors (HHV-6A: 1 IU/mL = 0.35 copies/mL, HHV-6B: 1 IU/mL = 0.96 copies/mL).

### Analytical and clinical performance evaluation

The analytical performance of the SMG assay was evaluated via determination of analytical sensitivity [LOD and lower limit of quantitation (LLOQ)], analytical specificity, linearity, and precision. The LOD and LLOQ of the SMG assay were assessed using heat-inactivated HHV-6A culture fluid (catalog no. 0810529CFHI) purchased from Zeptometrix (Buffalo, NY, USA) and the 1st World Health Organization (WHO) International Standard for HHV-6B DNA (NIBSC code: 15/266). These materials were serially diluted in HHV-6A/B-negative whole blood from a healthy volunteer, and 24 replicates per dilution level were tested. The analytical specificity of the SMG assay was assessed using 25 microorganisms (Table S1). Plasmids containing the HHV-6A and HHV-6B target sequences used for determination of linearity and precision of the SMG assay were obtained from Hwainmedics. Linearity was determined by analyzing a 10-fold dilution series of plasmids spiked in HHV-6A/B-negative whole blood, ranging from 3 to 9 log_10_ copies/mL, and nine replicates were tested for each concentration. Linear, second- and third-order polynomial regression fits were assessed. The precision of the SMG assay, i.e., intra- and inter-assay variabilities, was determined by analyzing the same 10-fold dilution series used in the linearity study. Intra-assay variability was determined by triplicate testing within the same run, while inter-assay variability was determined from three independent runs on different days. The clinical performance of the SMG assay was assessed and compared with that of the RealStar assay using 207 clinical specimens.

### Statistical analysis

The LOD was determined using probit regression analysis. The LLOQ was determined as the lowest concentration equal to or greater than the LOD and meeting the requirement for total analytical error (TAE), calculated as bias +2 × standard deviation, which is ≤0.5 log_10_ copies/mL. Simple linear regression was used to assess the relationship between nominal and measured viral loads, and linearity was considered acceptable if the coefficient of determination (*R*^2^) was greater than 0.98. Intra- and inter-assay variabilities were assessed by calculating the coefficient of variation (CV). The clinical sensitivity and specificity of the SMG and RealStar assays were calculated using in-house PCR and sequencing as a reference standard. The level of agreement between qualitative results provided by the two assays was determined by positive percent agreement (PPA), negative percent agreement (NPA), and Cohen’s kappa coefficient (*κ*). The level of agreement between viral loads measured by the two assays was analyzed using Bland-Altman plots, and mean differences and 95% limits of agreement (LOAs) were calculated. The correlation between viral loads measured by the two assays was analyzed using Passing-Bablok regression, and Spearman correlation coefficients (*ρ*) were calculated. Statistical analysis was conducted using R software (v.4.3.0), and data visualization was carried out using ggplot2 package in R software.

## RESULTS

### Analytical performance

**Fig 1:**
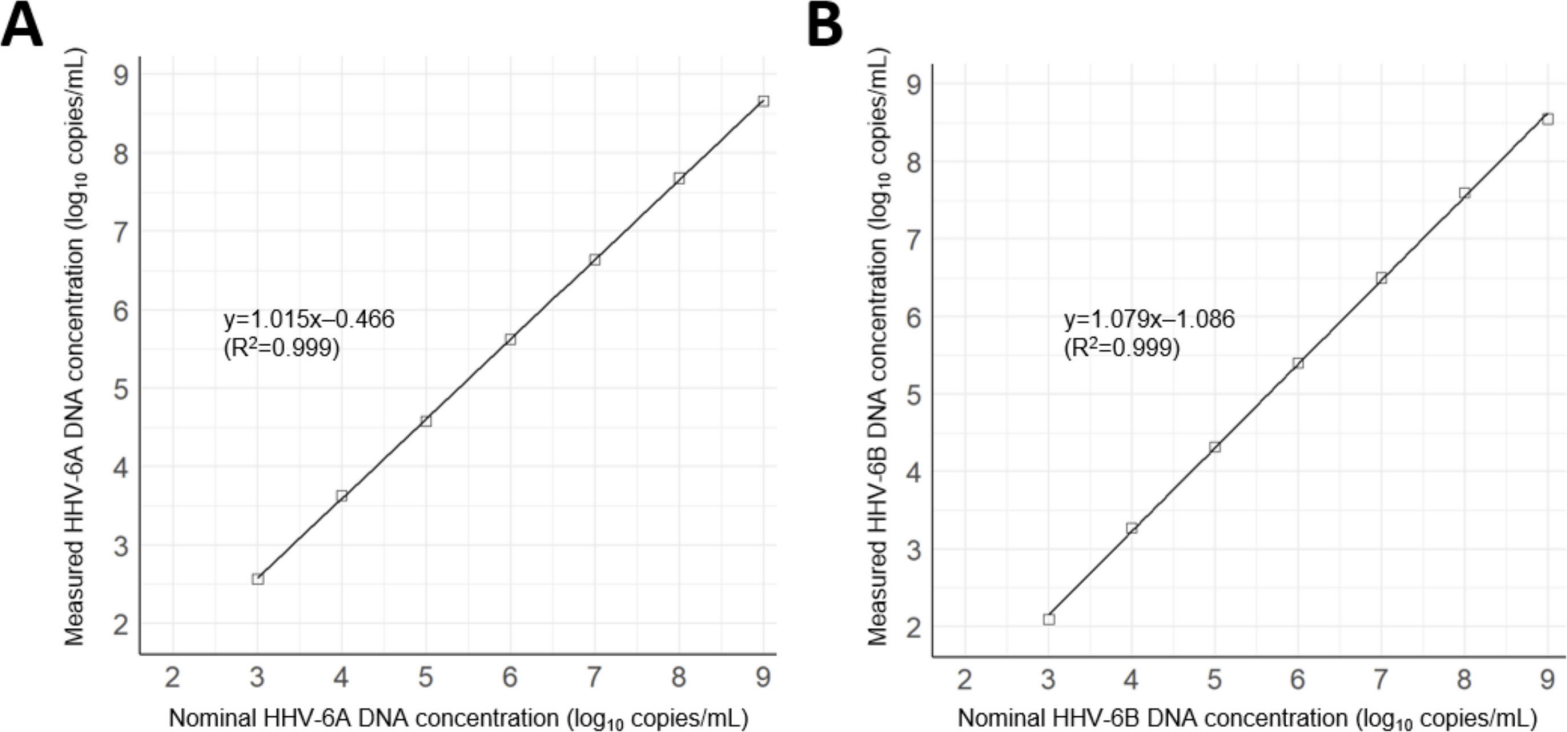
Linear ranges of the SMG assay for HHV-6A DNA (**A**) and HHV-6B DNA (**B**). The assay is linear over a range of 3–9 log_10_ copies/mL. Based on the LLOQ (3.40 log_10_ copies/mL), the linear range is 3.40–9.00 log_10_ copies/mL. The solid lines indicate linear regression lines.

The LOD of the SMG assay was 2.92 log_10_ copies/mL (95% CI: 2.84–3.04 log_10_ copies/mL) for HHV-6A DNA and 2.88 log_10_ copies/mL (95% CI: 2.80–3.00 log_10_ copies/mL) for HHV-6B DNA (Table 1). The LLOQ of the SMG assay was 3.40 log_10_ copies/mL for both HHV-6A and HHV-6B DNA. This was due to the TAE at this concentration being less than 0.5 log_10_ copies/mL (0.46 log_10_ copies/mL for HHV-6A DNA and 0.49 log_10_ copies/mL HHV-6B DNA). In the regression analysis, the first-order equation provided the best fit. Linearity was acceptable within the concentration range evaluated (3.00–9.00 log_10_ copies/mL) with an *R*^2^ of 0.999 for both HHV-6A and HHV-6B DNA. Based on the LLOQ (3.40 log_10_ copies/mL), the linear range of the SMG assay was determined to be 3.40–9.00 log_10_ copies/mL (Fig. 1). Intra- and inter-assay variabilities expressed as % CV were below 5% at concentrations ranging from 4 to 9 log_10_ copies/mL for both HHV-6A and HHV-6B DNA. However, at 3 log_10_ copies/mL, which is the concentration between the LOD and LLOQ of the SMG assay, intra- and inter-assay variabilities were greater than 5% for both viruses (Table 2). In the analytical specificity evaluation, the SMG assay did not exhibit cross-reactivity with any of the 25 microorganisms included in the specificity panel (Table S1).

**TABLE 1:** Analytical sensitivity evaluation results of the SMG assay^*a*^

Nominal DNA load (log_10_ copies/mL)	HHV-6A			HHV-6B		
	Mean DNA load (log_10_ copies/mL)	SD	Hit rate (%) (no. of positives/replicates)	Mean DNA load (log_10_ copies/mL)	SD	Hit rate (%) (no. of positives/replicates)
3.70	3.48	0.20	100 (24/24)	3.65	0.12	100 (24/24)
3.40	3.26	0.16	100 (24/24)	3.31	0.20	100 (24/24)
3.00	2.98	0.25	100 (24/24)	3.01	0.28	100 (24/24)
2.70	2.51	0.21	54.2 (13/24)	2.39	0.44	66.7 (16/24)
2.40	2.32	0.37	37.5 (9/24)	2.75	0.24	25.0 (6/24)
2.00	2.15	0.36	16.7 (4/24)	1.87	0.58	16.7 (4/24)
Probit LOD (95% CI)			2.92 (2.84–3.04)			2.88 (2.80–3.00)

^*a*^ CI, confidence interval; LOD, limit of detection; SD, standard deviation.

**TABLE 2:** Intra- and inter-assay variability of the SMG assay

Nominal DNA load (log_10_ copies/mL)	HHV-6A		HHV-6B	
	Mean DNA load (log_10_ copies/mL)	% CV^*a*^	Mean DNA load (log_10_ copies/mL)	% CV
Intra-assay variability (*n* = 3, 3 replicates per run × 1 run)				
9	8.53	0.18	8.64	0.24
8	7.53	0.70	7.50	0.28
7	6.53	0.72	6.44	0.80
6	5.57	1.20	5.35	2.01
5	4.60	1.03	4.32	2.63
4	3.68	2.22	3.30	1.15
3	2.72	7.52	2.09	9.42
Inter-assay variability (*n* = 9, 3 replicates per run × 3 runs)				
9	8.66	1.33	8.55	2.71
8	7.68	1.85	7.60	1.23
7	6.64	1.61	6.50	1.45
6	5.62	1.27	5.41	1.46
5	4.58	1.36	4.32	0.66
4	3.63	1.60	3.27	1.03
3	2.60	5.38	2.09	18.00

^*a*^ CV, coefficient of variation.

### Clinical performance

When using in-house PCR and sequencing as a reference standard, the clinical sensitivity and specificity of the SMG and RealStar assays are shown in Table 3. The SMG and RealStar assays yielded sensitivities of ≥96.9% and ≥90.6% for both HHV-6A and HHV-6B DNA, respectively. The specificities of the two assays were all 100% for both HHV-6A and HHV-6B DNA. The agreement between qualitative results obtained with the SMG and RealStar assays is shown in Table 4. The two assays exhibited almost perfect agreement with kappa coefficients of 1.00 (95% CI: 1.00–1.00) and 0.95 (95% CI: 0.91–1.00) for HHV-6A and HHV-6B DNA, respectively. Among the 51 specimens confirmed as HHV-6A positive by in-house PCR and sequencing, 1 was negative by both the SMG and RealStar assays. Among the 64 specimens confirmed as HHV-6B positive by in-house PCR and sequencing, 6 were negative by the RealStar assay, 4 of which were detected at concentrations lower than the LLOQ by the SMG assay (Tables 3 and 4). To rule out DNA degradation, these specimens were retested by in-house PCR and sequencing, which gave the same results as were obtained from routine HHV-6 testing using this method.

**TABLE 3:** Clinical sensitivity and specificity of the SMG and RealStar assays compared to in-house PCR and sequencing

qPCR assay	Target		In-house PCR and sequencing		Sensitivity (%) (95% CI)^*a*^	Specificity (%) (95% CI)
			Positive	Negative		
SMG assay	HHV-6A	Positive	50	0	98.0 (89.6%–100%)	100 (97.7%–100%)
		Negative	1	156		
	HHV-6B	Positive	62	0	96.9 (89.2%–99.6%)	100 (97.7%–100%)
		Negative	2	143		
RealStar assay	HHV-6A	Positive	50	0	98.0 (89.6%–100%)	100 (97.5%–100%)
		Negative	1	156		
	HHV-6B	Positive	58	0	90.6 (80.7%–96.5%)	100 (97.5%–100%)
		Negative	6	143		

^*a*^ CI, confidence interval.

**TABLE 4:** Agreement between qualitative results obtained with the SMG and RealStar assays^*a*^

Target	SMG assay	RealStar assay		PPA (%) (95% CI)	NPA (%) (95% CI)	Kappa (%) (95% CI)
		Positive	Negative			
HHV-6A	Positive	50	0	100 (92.9%–100.0%)	100 (97.7%–100.0%)	1.00 (1.00–1.00)
	Negative	0	157			
HHV-6B	Positive	58	4^*b*^	100 (93.8%–100.0%)	97.3 (93.3%–99.3%)	0.95 (0.91–1.00)
	Negative	0	145			

^*a*^ CI, confidence interval; NPA, negative percent agreement; PPA, positive percent agreement.

^*b*^ Four specimens negative for HHV-6B by the RealStar assay were detected at concentrations lower than the LLOQ by the SMG assay.

**Fig 2:**
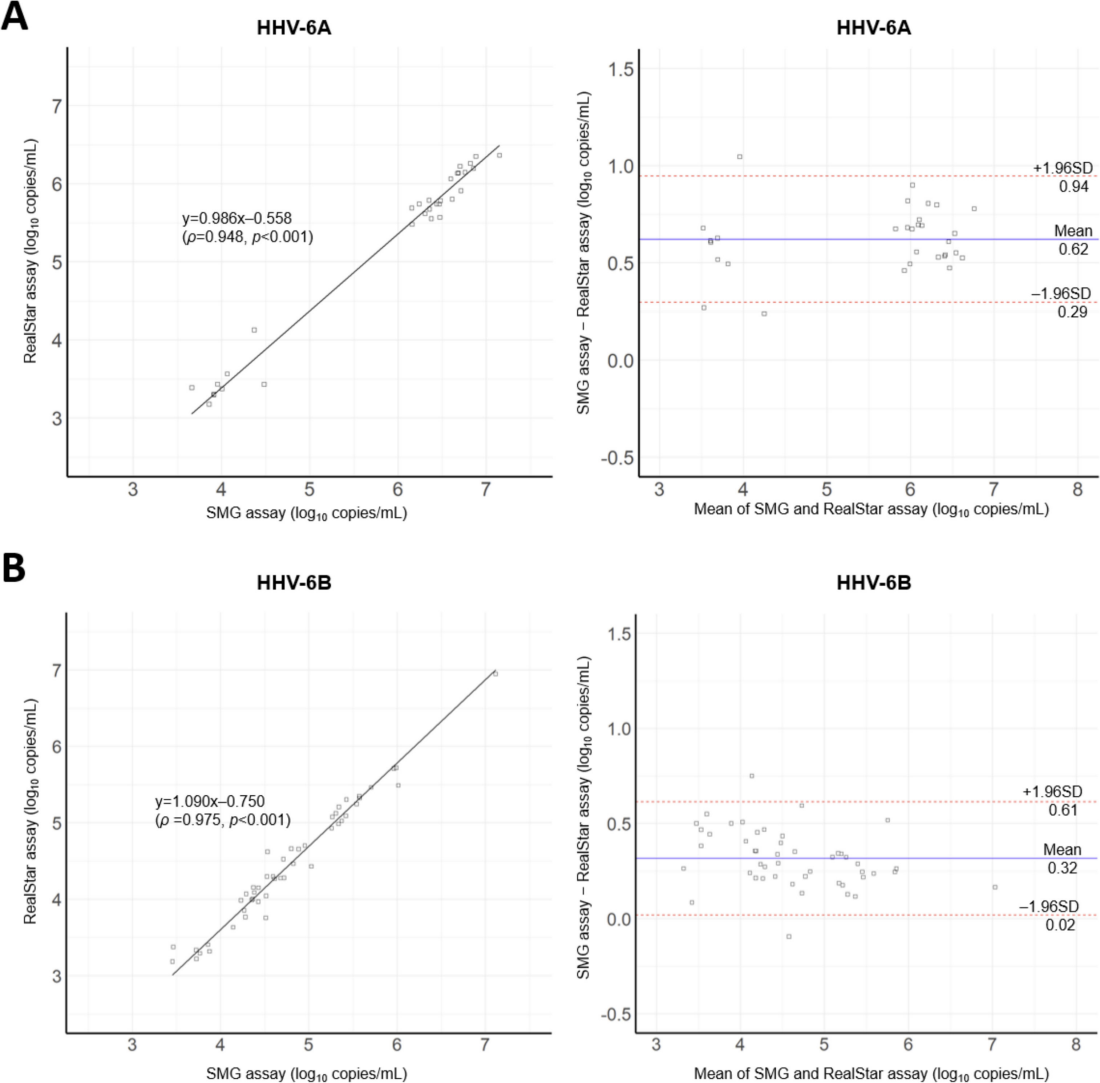
Comparison of viral loads measured by the SMG and RealStar assays. Passing-Bablok regression and Bland-Altman analyses of HHV-6A (**A**) and HHV-6B (**B**) viral loads measured by the two assays. In scatter plots, solid lines indicate Passing-Bablok regression lines. In Bland-Altman plots, solid and dashed lines indicate mean differences and 95% limits of agreement (mean differences ± 1.96 × standard deviation), respectively.

A total of 79 clinical specimens within the linear measurement range by both the SMG and RealStar assays were used for Passing-Bablok regression and Bland-Altman analyses. The concentrations of HHV-6A and HHV-6B DNA ranged from 3.86 to 7.15 log_10_ copies/mL (median 6.37 log_10_ copies/mL) and from 3.45 to 7.11 log_10_ copies/mL (median 4.64 log_10_ copies/mL), respectively, as measured by the SMG assay. Passing-Bablok regression analysis yielded correlation coefficients of 0.948 and 0.975 for HHV-6A and HHV-6B DNA, respectively. Bland-Altman analysis gave mean differences of 0.62 log_10_ copies/mL (95% LOA: 0.29–0.94 log_10_ copies/mL) and 0.32 log_10_ copies/mL (95% LOA: 0.02–0.61 log_10_ copies/mL) for HHV-6A and HHV-6B DNA, respectively (Fig. 2).

## DISCUSSION

With the increasing number of patients undergoing HSCT or SOT, the need for quantitative detection and differentiation of HHV-6A and HHV-6B DNA has become increasingly evident. In recent decades, several qPCR assays that can differentiate between HHV-6A and HHV-6B DNA, including the RealStar assay, have been developed and are routinely used in diagnostic laboratories (28, 31–33). In the present study, we evaluated the performance of the SMG assay, a newly developed qPCR assay, and compared the results to those of the RealStar assay. Our findings demonstrate that the SMG assay is highly sensitive and accurate and produces results comparable to those obtained using the RealStar assay.

In this study, the SMG assay showed high analytical sensitivity, with LODs of 2.92 and 2.88 log_10_ copies/mL for HHV-6A and HHV-6B DNA, respectively. According to the manufacturer’s package insert, the LODs of the RealStar assay were 3.17 and 3.13 log_10_ copies/mL elute for HHV-6A and HHV-6B DNA, respectively, suggesting that the analytical sensitivity of the SMG assay is comparable to that of the RealStar assay. The analytical specificity of the SMG assay was excellent, as no cross-reactivity was observed with 25 non-target microorganisms, including 7 viruses belonging to the Herpesviridae family (CMV, Epstein-Barr virus, human herpesvirus 7, human herpesvirus 8, herpes simplex viruses 1 and 2, and varicella zoster virus). In addition, the SMG assay exhibited a wide linear range from 3.40 to 9.00 log_10_ copies/mL for both HHV-6A and HHV-6B DNA. In individuals with iciHHV-6, HHV-6 DNA load in whole blood typically exceeds 6 log_10_ copies/mL, which is much higher than the values observed in patients with active infection (3–5 log_10_ copies/mL) (1, 36, 37). The wide linear range of the SMG assay enables reliable differentiation between active infection and iciHHV-6, preventing individuals with iciHHV-6 from being misdiagnosed with active infection and receiving unnecessary treatment. However, individuals with iciHHV-6 may have an HHV-6 DNA load of <5.5 log_10_ copies/mL in whole blood when the white blood cell count is significantly decreased (1, 37). Individuals without iciHHV-6 may also transiently have an HHV-6 DNA load of >5.5 log_10_ copies/mL in whole blood, albeit rarely (37). For these reasons, determination of the ratio of HHV-6 DNA to cellular DNA using qPCR or droplet digital PCR or detection of HHV-6 DNA integrated into human chromosomes using fluorescence *in situ* hybridization should be used as confirmatory testing (1, 24, 37). In this study, the SMG assay showed good precision, with intra- and inter-assay variabilities of less than 5% at concentrations ranging from 4 to 9 log_10_ copies/mL for both HHV-6A and HHV-6B DNA. However, at 3 log_10_ copies/mL, which is the concentration between LOD and LLOQ of the SMG assay, intra- and inter-assay variabilities of greater than 5% were noted. As HHV-6 DNA load of 3 log_10_ copies/mL in whole blood is considered as a tentative threshold to distinguish between latent and active infections (1, 38), the low precision of the SMG assay at this concentration can lead to misdiagnosis and inappropriate treatment decisions.

When using in-house PCR and sequencing as a reference standard, the SMG and RealStar assays showed high sensitivity and specificity in detecting HHV-6A and HHV-6B DNA in whole blood. There were seven specimens positive by in-house PCR and sequencing but not detected or detected at concentrations lower than the LLOQ by the SMG or RealStar assays. A possible explanation for this discrepancy might be the presence of mutations in the primer/probe binding sites of the SMG and RealStar assays, as these mutations may result in underquantification or false-negative results for HHV-6 DNA. Since the clinical performance of the SMG and RealStar assays may vary, depending on the prevalence of HHV-6 strains harboring these mutations in a local population, local validation studies are required before use in diagnostic laboratories. Although the SMG assay, unlike the RealStar assay, was not calibrated against the 1st WHO International Standard for HHV-6B DNA, the quantitative agreement between the two assays was good. The correlation coefficients between viral loads measured by the two assays were 0.948 and 0.977, with mean differences of 0.62 and 0.32 log_10_ copies/mL for HHV-6A and HHV-6B DNA, respectively. The WHO International Standard for HHV-6A DNA has yet to be established, and it remains a major barrier to the standardization of qPCR assays for the quantitative detection and differentiation of HHV-6A and HHV-6B DNA.

A major limitation of our study is that the performance characteristics of the SMG and RealStar assays were not established for specimen types other than whole blood. In addition to whole blood, various specimen types, such as plasma, serum, cerebrospinal fluid, and bronchoalveolar lavage, can be used for diagnosis of active infection (1). The performance characteristics of the SMG and RealStar assays for these specimen types should be established in further studies. Another limitation of our study is that archived DNA specimens were used to assess the clinical performance of the SMG and RealStar assays. Degradation of viral DNA may have occurred during long-term storage or the freeze-thaw process, which can be a factor in underestimating the clinical performance of the SMG and RealStar assays. Despite the use of archived DNA specimens, the SMG and RealStar assays showed high clinical sensitivity and specificity, and the viral loads obtained with the two assays were well correlated; however, further prospective studies are needed to accurately assess their clinical performance. Lastly, while in our clinical evaluation, we employed two extraction methods: MagNA Pure 96 and KingFisher Flex. We did not investigate how each extraction method influenced the performance of the SMG and RealStar assays. This will be the focus of future studies.

In conclusion, the SMG assay demonstrated excellent analytical sensitivity and specificity, precision, and linearity. Furthermore, the viral loads measured by the SMG assay were highly correlated with those measured by the RealStar assay routinely used in diagnostic laboratories. Taken together, the SMG assay is a useful diagnostic tool for quantitative detection and differentiation of HHV-6A and HHV-6B DNA in whole blood.

## SUPPLEMENTAL MATERIAL

Table S1 spectrum.04249-23-s0001.pdf Analytical specificity evaluation results of the SMG assay.

ASM does not own the copyrights to Supplemental Material that may be linked to, or accessed through, an article. The authors have granted ASM a non-exclusive, world-wide license to publish the Supplemental Material files. Please contact the corresponding author directly for reuse.

## References

[1] Agut H, Bonnafous P, Gautheret-Dejean A. 2015. Laboratory and clinical aspects of human herpesvirus 6 infections. Clin Microbiol Rev 28:313–335. doi:10.1128/CMR.00122-14 25762531 PMC4402955

[2] Braun DK, Dominguez G, Pellett PE. 1997. Human herpesvirus 6. Clin Microbiol Rev 10:521–567. doi:10.1128/CMR.10.3.521 9227865 PMC172933

[3] Kim F, Reichman V, Hooven TA. 2020. Human herpesvirus-6 meningitis in a premature infant with fevers: a case and literature review. Clin Med Insights Case Rep 13:1179547620912952. doi:10.1177/1179547620912952 32341669 PMC7169356

[4] Campadelli-Fiume G, Mirandola P, Menotti L. 1999. Human herpesvirus 6: an emerging pathogen. Emerg Infect Dis 5:353–366. doi:10.3201/eid0503.990306 10341172 PMC2640789

[5] Arbuckle JH, Medveczky MM, Luka J, Hadley SH, Luegmayr A, Ablashi D, Lund TC, Tolar J, De Meirleir K, Montoya JG, Komaroff AL, Ambros PF, Medveczky PG. 2010. The latent human herpesvirus-6A genome specifically integrates in telomeres of human chromosomes in vivo and in vitro. Proc Natl Acad Sci U S A 107:5563–5568. doi:10.1073/pnas.0913586107 20212114 PMC2851814

[6] Arbuckle JH, Pantry SN, Medveczky MM, Prichett J, Loomis KS, Ablashi D, Medveczky PG. 2013. Mapping the telomere integrated genome of human herpesvirus 6A and 6B. Virology 442:3–11. doi:10.1016/j.virol.2013.03.030 23648233 PMC3696530

[7] Tanaka-Taya K, Sashihara J, Kurahashi H, Amo K, Miyagawa H, Kondo K, Okada S, Yamanishi K. 2004. Human herpesvirus 6 (HHV-6) is transmitted from parent to child in an integrated form and characterization of cases with chromosomally integrated HHV-6 DNA. J Med Virol 73:465–473. doi:10.1002/jmv.20113 15170644

[8] Leong HN, Tuke PW, Tedder RS, Khanom AB, Eglin RP, Atkinson CE, Ward KN, Griffiths PD, Clark DA. 2007. The prevalence of chromosomally integrated human herpesvirus 6 genomes in the blood of UK blood donors. J Med Virol 79:45–51. doi:10.1002/jmv.20760 17133548

[9] Ward KN, Leong HN, Thiruchelvam AD, Atkinson CE, Clark DA. 2007. Human herpesvirus 6 DNA levels in cerebrospinal fluid due to primary infection differ from those due to chromosomal viral integration and have implications for diagnosis of encephalitis. J Clin Microbiol 45:1298–1304. doi:10.1128/JCM.02115-06 17229866 PMC1865851

[10] Hall CB, Caserta MT, Schnabel K, Shelley LM, Marino AS, Carnahan JA, Yoo C, Lofthus GK, McDermott MP. 2008. Chromosomal integration of human herpesvirus 6 is the major mode of congenital human herpesvirus 6 infection. Pediatrics 122:513–520. doi:10.1542/peds.2007-2838 18762520

[11] Tweedy J, Spyrou MA, Hubacek P, Kuhl U, Lassner D, Gompels UA. 2015. Analyses of germline, chromosomally integrated human herpesvirus 6A and B genomes indicate emergent infection and new inflammatory mediators. J Gen Virol 96:370–389. doi:10.1099/vir.0.068536-0 25355130

[12] Yoshikawa T. 2004. Human herpesvirus 6 infection in hematopoietic stem cell transplant patients. Br J Haematol 124:421–432. doi:10.1046/j.1365-2141.2003.04788.x 14984492

[13] Wang LR, Dong LJ, Zhang MJ, Lu DP. 2006. The impact of human herpesvirus 6B reactivation on early complications following allogeneic hematopoietic stem cell transplantation. Biol Blood Marrow Transplant 12:1031–1037. doi:10.1016/j.bbmt.2006.06.001 17067909

[14] Wang L-R, Dong L-J, Zhang M-J, Lu D-P. 2008. Correlations of human herpesvirus 6B and CMV infection with acute GVHD in recipients of allogeneic haematopoietic stem cell transplantation. Bone Marrow Transplant 42:673–677. doi:10.1038/bmt.2008.238 18695666

[15] Lautenschlager I, Razonable RR. 2012. Human herpesvirus-6 infections in kidney, liver, lung, and heart transplantation: review. Transpl Int 25:493–502. doi:10.1111/j.1432-2277.2012.01443.x 22356254

[16] Zerr DM, Boeckh M, Delaney C, Martin PJ, Xie H, Adler AL, Huang ML, Corey L, Leisenring WM. 2012. HHV-6 reactivation and associated sequelae after hematopoietic cell transplantation. Biol Blood Marrow Transplant 18:1700–1708. doi:10.1016/j.bbmt.2012.05.012 22641196 PMC3439599

[17] Sedlak RH, Hill JA, Nguyen T, Cho M, Levin G, Cook L, Huang ML, Flamand L, Zerr DM, Boeckh M, Jerome KR. 2016. Detection of human herpesvirus 6B (HHV-6B) reactivation in hematopoietic cell transplant recipients with inherited chromosomally integrated HHV-6A by droplet digital PCR. J Clin Microbiol 54:1223–1227. doi:10.1128/JCM.03275-15 26888901 PMC4844705

[18] Ogata M, Oshima K, Ikebe T, Takano K, Kanamori H, Kondo T, Ueda Y, Mori T, Hashimoto H, Ogawa H, Eto T, Ueki T, Miyamoto T, Ichinohe T, Atsuta Y, Fukuda T. 2017. Clinical characteristics and outcome of human herpesvirus-6 encephalitis after allogeneic hematopoietic stem cell transplantation. Bone Marrow Transplant 52:1563–1570. doi:10.1038/bmt.2017.175 28783148

[19] Raouf MME, Ouf NM, Elsorady MAS, Ghoneim FM. 2023. Human herpesvirus-6 in hematopoietic stem cell transplant recipients: a prospective cohort study in Egypt. Virol J 20:20. doi:10.1186/s12985-023-01980-w 36739398 PMC9899109

[20] Dominguez G, Dambaugh TR, Stamey FR, Dewhurst S, Inoue N, Pellett PE. 1999. Human herpesvirus 6B genome sequence: coding content and comparison with human herpesvirus 6A. J Virol 73:8040–8052. doi:10.1128/JVI.73.10.8040-8052.1999 10482553 PMC112820

[21] Adams MJ, Carstens EB. 2012. Ratification vote on taxonomic proposals to the international committee on taxonomy of viruses. Arch Virol 157:1411–1422. doi:10.1007/s00705-012-1299-6 22481600 PMC7086667

[22] Ablashi D, Agut H, Alvarez-Lafuente R, Clark DA, Dewhurst S, DiLuca D, Flamand L, Frenkel N, Gallo R, Gompels UA, Höllsberg P, Jacobson S, Luppi M, Lusso P, Malnati M, Medveczky P, Mori Y, Pellett PE, Pritchett JC, Yamanishi K, Yoshikawa T. 2014. Classification of HHV-6A and HHV-6B as distinct viruses. Arch Virol 159:863–870. doi:10.1007/s00705-013-1902-5 24193951 PMC4750402

[23] Pellett Madan R, Hand J, AST Infectious Diseases Community of Practice. 2019. Human herpesvirus 6, 7, and 8 in solid organ transplantation: guidelines from the American society of transplantation infectious diseases community of practice. Clin Transplant 33:e13518. doi:10.1111/ctr.13518 30844089

[24] Ward KN, Hill JA, Hubacek P, de la Camara R, Crocchiolo R, Einsele H, Navarro D, Robin C, Cordonnier C, Ljungman P, 2017 European Conference on Infections in Leukaemia (ECIL). 2019. Guidelines from the 2017 European conference on infections in leukaemia for management of HHV-6 infection in patients with hematologic malignancies and after hematopoietic stem cell transplantation. Haematologica 104:2155–2163. doi:10.3324/haematol.2019.223073 31467131 PMC6821622

[25] Caserta MT, Hall CB, Schnabel K, Lofthus G, Marino A, Shelley L, Yoo C, Carnahan J, Anderson L, Wang H. 2010. Diagnostic assays for active infection with human herpesvirus 6 (HHV-6). J Clin Virol 48:55–57. doi:10.1016/j.jcv.2010.02.007 20211581 PMC2855742

[26] Takano K, Ogata M, Kawano R, Satou T, Nashimoto Y, Shirao K. 2018. Comparison of HHV-6 DNA detection in plasma and whole blood in allogeneic hematopoietic stem cell transplant recipients: frequent false-positive results for active HHV-6 infection using whole blood samples. Int J Hematol 108:535–542. doi:10.1007/s12185-018-2498-z 30014227

[27] Obeid M, Gakhal I, McDonald PJ. 2021. Persistent viremia in an immunocompetent patient with inherited chromosomally integrated HHV-6B. Access Microbiol 3:000256. doi:10.1099/acmi.0.000256 34888484 PMC8650848

[28] Reddy S, Manna P. 2005. Quantitative detection and differentiation of human herpesvirus 6 subtypes in bone marrow transplant patients by using a single real-time polymerase chain reaction assay. Biol Blood Marrow Transplant 11:530–541. doi:10.1016/j.bbmt.2005.04.010 15983553

[29] Flamand L, Gravel A, Boutolleau D, Alvarez-Lafuente R, Jacobson S, Malnati MS, Kohn D, Tang YW, Yoshikawa T, Ablashi D. 2008. Multicenter comparison of PCR assays for detection of human herpesvirus 6 DNA in serum. J Clin Microbiol 46:2700–2706. doi:10.1128/JCM.00370-08 18550745 PMC2519497

[30] Wada K, Mizoguchi S, Ito Y, Kawada J-I, Yamauchi Y, Morishima T, Nishiyama Y, Kimura H. 2009. Multiplex real-time PCR for the simultaneous detection of herpes simplex virus, human herpesvirus 6, and human herpesvirus 7. Microbiol Immunol 53:22–29. doi:10.1111/j.1348-0421.2008.00090.x 19161554

[31] de Pagter PJ, Schuurman R, de Vos NM, Mackay W, van Loon AM. 2010. Multicenter external quality assessment of molecular methods for detection of human herpesvirus 6. J Clin Microbiol 48:2536–2540. doi:10.1128/JCM.01145-09 20147642 PMC2897485

[32] Cassina G, Russo D, De Battista D, Broccolo F, Lusso P, Malnati MS. 2013. Calibrated real-time polymerase chain reaction for specific quantitation of HHV-6A and HHV-6B in clinical samples. J Virol Methods 189:172–179. doi:10.1016/j.jviromet.2013.01.018 23391825

[33] Yip CCY, Sridhar S, Cheng AKW, Fung AMY, Cheng VCC, Chan KH, Yuen KY. 2017. Comparative evaluation of a laboratory developed real-time PCR assay and the RealStar HHV-6 PCR Kit for quantitative detection of human herpesvirus 6. J Virol Methods 246:112–116. doi:10.1016/j.jviromet.2017.05.001 28476346

[34] Zheng Y, Zhao Y, Wang Y, Rao J. 2021. A multiplex real-time PCR quantitation of human herpesvirus-6, 7, 8 viruses: application in blood transfusions. Virol J 18:38. doi:10.1186/s12985-021-01510-6 33602271 PMC7891017

[35] Mah J, Huang C, Sahoo MK, Pinsky BA. 2023. Evaluation of an automated system for the quantitation of human herpesvirus-6 DNA from clinical specimens. Pract Lab Med 36:e00329. doi:10.1016/j.plabm.2023.e00329 37649537 PMC10462668

[36] Ward KN, Leong HN, Nacheva EP, Howard J, Atkinson CE, Davies NWS, Griffiths PD, Clark DA. 2006. Human herpesvirus 6 chromosomal integration in immunocompetent patients results in high levels of viral DNA in blood, sera, and hair follicles. J Clin Microbiol 44:1571–1574. doi:10.1128/JCM.44.4.1571-1574.2006 16597897 PMC1448653

[37] Pellett PE, Ablashi DV, Ambros PF, Agut H, Caserta MT, Descamps V, Flamand L, Gautheret-Dejean A, Hall CB, Kamble RT, et al.. 2012. Chromosomally integrated human herpesvirus 6: questions and answers. Rev Med Virol 22:144–155. doi:10.1002/rmv.715 22052666 PMC3498727

[38] Boutolleau D, Fernandez C, André E, Imbert-Marcille BM, Milpied N, Agut H, Gautheret-Dejean A. 2003. Human herpesvirus (HHV)-6 and HHV-7: two closely related viruses with different infection profiles in stem cell transplantation recipients. J Infect Dis 187:179–186. doi:10.1086/367677 12552442

