# Supplementary material for: Performance evaluation of the SMG HHV-6 Q Real-Time PCR Kit for quantitative detection and differentiation of human herpesvirus 6A and 6B
Source: Microbiol Spectr. 2024 Mar 7;12(4):e04249-23. doi: 10.1128/spectrum.04249-23 (PMC10986541; doi:10.1128/spectrum.04249-23)
Supplement: Table S1 — Analytical specificity evaluation results of the SMG assay. [file spectrum.04249-23-s0001.pdf]

Table S1. Analytical specificity evaluation results of the SMG assay

Microorganism	Source (code number)[a]	Result
BK virus	NIBSC (14/212)	Negative
Cytomegalovirus	NIBSC (09/162)	Negative
Epstein-Barr virus	NIBSC (09/260)	Negative
Hepatitis A Virus	KBPV (VR-78)	Negative
Hepatitis B Virus	NIBSC (10/266)	Negative
Hepatitis C Virus	NIBSC (18/184)	Negative
Herpes simplex virus 1	KBPV (VR-83)	Negative
Herpes simplex virus 2	KBPV (VR-84)	Negative
Human herpesvirus 7	Zeptometrix (0810071CFHI)	Negative
Human herpesvirus 8	Zeptometrix (0810104CFHI)	Negative
JC virus	NIBSC (14/114)	Negative
Parvovirus B19	NIBSC (12/208)	Negative
Varicella-Zoster virus	NIBSC (19/164)	Negative
Gardnerella vaginalis	ATCC (49145D-5)	Negative
Haemophilus influenzae	KCCM (42099)	Negative
Mycobacterium avium	Clinical isolate	Negative
Mycobacterium tuberculosis	Clinical isolate	Negative
Mycoplasma genitalium	ATCC (33530D)	Negative
Mycoplasma hominis	ATCC (23114D)	Negative
Neisseria gonorrhoeae	ATCC (700825D-5)	Negative
Streptococcus mitis	KCTC (5650)	Negative
Streptococcus mutans	KCCM (40105)	Negative
Streptococcus pneumoniae	ATCC (33400D-5)	Negative
Trichomonas vaginalis	ATCC (30001D)	Negative
Candida albicans	ATCC (MYA-2876D-5)	Negative

[a]NIBSC, National Institute for Biological Standards and Control; KBPV, Korea Bank for Pathogenic Viruses; ATCC, American Type Culture Collection; KCCM, Korean Culture Center of Microorganisms; KCTC, Korean Collection for Type Cultures.
